# Does technology-based interventions in psychosis improved functioning and quality of life? A systematic review and meta-analysis

**DOI:** 10.1192/j.eurpsy.2022.636

**Published:** 2022-09-01

**Authors:** C. Morales-Pillado, T. Sanchez-Gutierrez, B. Fernandez-Castilla, S. Barbeito, E. Gonzalez-Fraile, A. Calvo

**Affiliations:** 1 Universidad Internacional de La Rioja, Faculty Of Health Sciences, Madrid, Spain; 2 University of Leuven, Faculty Of Psychology And Educational Sciences, Leuven, Belgium

**Keywords:** Psychosis, Technology-based interventions, Mobile interventions, schizophrénia

## Abstract

**Introduction:**

Technology-based interventions (TBIs), including computer and Internet-based interventions, mobile interventions, health applications, social media interventions, and interventions using technological devices, could become a useful, effective, accessible, and cost-effective approach (Berry et al., 2016; Firth, 2016) to complement conventional interventions for psychosis

**Objectives:**

to compare TBIs with conventional interventions for psychosis, focusing mainly on functioning and quality of life.

**Methods:**

The systematic review preceding this work was based on 58 RCT of TBIs for psychosis. We selected the studies that analyzed functioning (N = 23) and quality of life (N = 15). We calculated the standardized mean change (SMC) and applied a three-level model because there were several effect sizes within the same study.

**Results:**

There were significant differences between TBIs and conventional interventions for functioning (d = 0.25, SE = 0.09, z = 2.72, p = <.01), but not for quality of life (d = 0.14, SE = 0.08, z = 1.78, p = .076) in patients with psychosis.

**Conclusions:**

On average, patients who received TBIs performed better in functioning, but not in quality of life. Functioning is impaired in patients with psychosis, so TBIs should be considered a complement and efficacious intervention, highlighting the power of these type of interventions in improving some outcomes.

**Disclosure:**

No significant relationships.

